# Chicken Juice Enhances Surface Attachment and Biofilm Formation of Campylobacter jejuni

**DOI:** 10.1128/AEM.02614-14

**Published:** 2014-11

**Authors:** Helen L. Brown, Mark Reuter, Louise J. Salt, Kathryn L. Cross, Roy P. Betts, Arnoud H. M. van Vliet

**Affiliations:** a Institute of Food Research, Norwich, United Kingdom; b Campden BRI, Chipping Campden, Gloucestershire, United Kingdom

## Abstract

The bacterial pathogen Campylobacter jejuni is primarily transmitted via the consumption of contaminated foodstuffs, especially poultry meat. In food processing environments, C. jejuni is required to survive a multitude of stresses and requires the use of specific survival mechanisms, such as biofilms. An initial step in biofilm formation is bacterial attachment to a surface. Here, we investigated the effects of a chicken meat exudate (chicken juice) on C. jejuni surface attachment and biofilm formation. Supplementation of brucella broth with ≥5% chicken juice resulted in increased biofilm formation on glass, polystyrene, and stainless steel surfaces with four C. jejuni isolates and one C. coli isolate in both microaerobic and aerobic conditions. When incubated with chicken juice, C. jejuni was both able to grow and form biofilms in static cultures in aerobic conditions. Electron microscopy showed that C. jejuni cells were associated with chicken juice particulates attached to the abiotic surface rather than the surface itself. This suggests that chicken juice contributes to C. jejuni biofilm formation by covering and conditioning the abiotic surface and is a source of nutrients. Chicken juice was able to complement the reduction in biofilm formation of an aflagellated mutant of C. jejuni, indicating that chicken juice may support food chain transmission of isolates with lowered motility. We provide here a useful model for studying the interaction of C. jejuni biofilms in food chain-relevant conditions and also show a possible mechanism for C. jejuni cell attachment and biofilm initiation on abiotic surfaces within the food chain.

## INTRODUCTION

Infection by Campylobacter species is a global public health concern, estimated to affect 1% of the population in the developed world annually (1). Campylobacter jejuni is the most common cause of human Campylobacter infection, representing up to 90% of isolates from clinical cases (2). Infection with C. jejuni is also linked to severe postinfectious sequelae, such as Guillain-Barré syndrome and reactive arthritis (3–6). This combination of high disease load and severe postinfectious complications makes C. jejuni infection a significant economic and disease burden in many countries worldwide.

The major transmission route for C. jejuni is thought to be via contaminated food stuffs, with poultry meat being the main source of infection in urban cases. Sampling of chicken meat from supermarkets showed that up to 70% of meat is contaminated with C. jejuni (7). In laboratory conditions, C. jejuni is a fastidious organism that requires a temperature of 34 to 44°C and microaerobic conditions for growth. However, during transmission through the food chain it encounters stresses, such as changes in temperature, exposure to aerobic conditions, and lack of nutrients. Significant advances have been made in the understanding of C. jejuni stress responses; however, there is still a lack of understanding of how these work together to allow survival of C. jejuni in the human food chain. One possible contributor to this survival is the ability of C. jejuni to form biofilms (8–11).

Biofilms are commonly defined as attached bacterial colonies of either single or multiple species, encased in an extracellular matrix (11). Biofilms support the survival of bacteria in suboptimal conditions and increase resistance to disinfectants, antimicrobials, and antibiotics (10, 12). To date, it is estimated that 99% of bacteria can grow in biofilms, and it is has been suggested that for the majority of bacteria, biofilms are the normal mode of existence (13). C. jejuni has been shown to form a monospecies biofilm (8–11, 14, 15) and can also integrate into preexisting biofilms (16).

A serious problem in food processing areas is insufficient or ineffective removal of organic material. Spilled foodstuffs or runoff from carcass eviscerations contain a complex blend of carbohydrates, proteins, lipids, and sugars (17), providing an ideal medium for bacteria to thrive and survive. A build-up of these organic materials on a surface is here referred to as a conditioning layer. Conditioning layers assist bacterial attachment to surfaces by altering the surface physicochemical properties and attracting the bacteria to the surface due to the increased nutrient availability (18, 19). One well-studied example of a conditioning layer is the oral pellicle, which assists in the attachment of bacterial species such as Streptococcus mutans to the tooth surface and contributes to subsequent periodontal disease (20). Surface conditioning layers have also been shown to be important for the initial attachment of food-borne pathogens; for example, Listeria monocytogenes survival rates increase when biological soil is present on stainless steel surfaces (21), and milk proteins are able to increase the attachment of Escherichia coli, L. monocytogenes, and Staphylococcus aureus to stainless steel (22).

To date, most studies on C. jejuni biofilms have been performed in laboratory conditions, which do not mimic the conditions encountered in the processing environment. It is important to ensure that studies are designed to allow accurate interpretation and extrapolation of laboratory-obtained results to the food industry (23). Various experimental systems have been used to mimic the conditions encountered by C. jejuni in the food chain. These models typically include the use of cooked or raw meat (24), modeling relevant packaging conditions (23), or the use materials relevant to the food chain such as stainless steel (25). One such model system is the “chicken juice” model (26). This model is based on the collection of exudate from defrosted, commercially obtained chicken carcasses, followed by supplementation or replacement of standard laboratory media with this sterile-filtered liquid. Supplementation of brucella broth with chicken juice resulted in increased survival of planktonic cells of C. jejuni following both chilled and frozen storage (26, 27).

We investigated here the effect of chicken juice on the attachment of C. jejuni to surfaces and subsequent biofilm formation. We show that in the presence of chicken juice, C. jejuni biofilm formation is increased and that this increase in biofilm levels is not simply due to increased cell numbers within the suspensions but to an increase in attachment to abiotic surfaces. We show that this increase in attachment is due to the ability of chicken juice to condition abiotic surfaces relevant to food processing environments.

## MATERIALS AND METHODS

### C. jejuni strains and growth conditions.

C. jejuni reference strains NCTC 11168 (28), 81116 (29), 81-176 (30), and RM1221 (31), an NCTC 11168 nonmotile (aflagellate) mutant (NCTC 11168 Δ*flaAB*) (10), and C. coli clinical isolate 15-537360 (32) were routinely cultured in a MACS-MG-1000 controlled atmosphere cabinet (Don Whitley Scientific) under microaerobic conditions (85% N_2_, 5% O_2_, 10% CO_2_) at 37°C. For growth on plates, strains were either grown on brucella agar or blood agar base (Becton Dickinson) with Skirrow supplements (10 μg/ml vancomycin, 5 μg/ml trimethoprim, 2.5 IU of polymyxin B). Broth culture was carried out in brucella broth (Becton Dickinson). An Innova 4230 (New Brunswick Scientific) incubator was used for aerobic culture at 37°C.

### Preparation of chicken juice.

Chicken juice (meat exudate) was prepared as described previously (26). Briefly, frozen whole chickens were purchased from four different United Kingdom supermarkets, with no significant differences observed between different supermarkets and different whole chicken (data not shown). The whole chickens were thawed overnight at room temperature, and the exudate was collected, centrifuged to remove debris, and sterilized by using a 0.2-μm-pore-size sterile polyethersulfone syringe filter (Millipore). Chicken juice was divided into aliquots and stored at −20°C until use. Chicken juice was diluted to various percentages (vol/vol) in brucella broth unless stated otherwise.

### Precoating of abiotic surfaces.

Sterile stainless steel coupons (stainless steel type 1.4301 according to European Standard EN 10088-1, with a type 2B finish according to European Standard EN 10088-2) were placed in a six-well polystyrene tissue culture plate (Corning) and incubated with 4 ml of brucella broth, brucella broth containing chicken juice, or 100% chicken juice. Likewise, sterile borosilicate glass test tubes (Corning) were incubated with 1 ml of brucella broth, brucella broth containing chicken juice, or 100% chicken juice. Samples were incubated overnight at 37°C in aerobic conditions to allow precoating. The medium was subsequently removed, and surfaces were washed with an equal volume of phosphate-buffered saline (PBS; 1 ml for test tubes, 4 ml for six-well plates with stainless steel coupons) and immediately used for biofilm assays using brucella broth.

### Campylobacter growth for biofilm assay.

Single-use glycerol stocks of C. jejuni were thawed, inoculated onto Skirrow plates and grown overnight at 37°C in microaerobic conditions (5% O_2_, 10% CO_2_, 85% N_2_). Cells from the Skirrow plate were used to inoculate brucella broth and incubated overnight shaking (37°C, microaerobic conditions). After overnight growth, cell cultures were adjusted to an *A*_600_ of 0.05 in brucella broth, brucella broth supplemented with 5% (vol/vol) chicken juice or 100% chicken juice. To allow biofilm formation, 1 ml of this solution was added to either a sterile borosilicate glass test tube (Corning) or a 24-well polystyrene tissue culture plate (Corning), or 3 ml was added to a six-well polystyrene tissue culture plate (Corning) containing a sterile stainless steel coupon. Tubes were incubated at 37°C under either microaerobic or atmospheric air conditions for 48 h before staining.

### Congo red staining.

A 0.1% (vol/vol) concentration of Congo red was added to brucella broth, brucella broth supplemented with 5% (vol/vol) chicken juice, or 100% chicken juice with or without C. jejuni, before static incubation at 37°C in microaerobic conditions for 48 h. At the end of the incubation period, the medium was removed from the tube before washing with 1 ml of PBS and drying at 37°C. Bound Congo red dye was dissolved by adding 20% acetone–80% ethanol, followed by incubation on a rocking platform for 15 min at room temperature. The level of dissolved dye was measured at a wavelength of 500 nm using a Biomate 5 spectrophotometer (Thermo Scientific).

### Crystal violet staining.

Biofilms were formed in brucella broth, brucella broth supplemented with 5% (vol/vol) chicken juice, or 100% chicken juice. After biofilm formation, the medium was removed from the test tubes before the samples were washed with water and dried at 60°C for 30 min. Next, 1 ml of 1% (wt/vol) crystal violet solution was added, and the tubes were further incubated on a rocker at room temperature for 30 min. The unbound dye was removed from the tubes by thorough washing in water, followed by drying at 37°C. Bound crystal violet was dissolved by adding 20% acetone–80% ethanol, followed by incubation on a rocking platform for 15 min at room temperature. The resulting dissolved dye was measured at a wavelength of 590 nm by using a Biomate 5 spectrophotometer (Thermo Scientific).

### TTC staining.

2,3,5-Triphenyltetrazolium chloride (TTC) staining was carried out as described previously (33). Briefly, cell suspensions were removed after 48 h of incubation, and the tubes were washed twice with 1 ml of sterile PBS. Then, 1.2 ml of brucella broth supplemented with 0.05% (wt/vol) TTC was added to each test tube before incubation at 37°C under microaerobic conditions for 72 h. After incubation, the TTC solution was removed, and the test tubes were air dried. Bound TTC dye was dissolved as described above using 20% acetone–80% ethanol, and the *A*_500_ of the solution was measured.

### Assessment of cell viability by culture.

To determine the number of viable cells in the planktonic fraction, the medium of biofilm experiments was 10-fold serially diluted in PBS, and 5 μl of each dilution was spotted onto brucella agar plates. After 2 days of growth, the dilution resulting in two or more colonies was recorded. Cell viability in biofilm assays was assessed upon initial addition of cultures into static culture, before washing and the addition of TTC-containing media and after incubation with TTC-containing media. The cell viability in growth assays was assessed every 2 h in the first 8 h of the experiment and every 24 h thereafter.

### Use of TTC as a growth indicator.

C. jejuni was grown as described above and diluted to an *A*_600_ of 0.05 in brucella broth supplemented with 0.05% TTC before incubation at 37°C under microaerobic conditions for 48 h (33). Formazan crystals were then dissolved by adding an equal volume of 20% acetone–80% ethanol and incubating the samples at room temperature for 30 min before centrifugation (20,000 × *g*, 10 min at room temperature). The *A*_500_ of the supernatant was then measured.

### SEM.

The biofilms were collected on Thermanox coverslips (Agar Scientific, Stansted, United Kingdom) and fixed with 2.5% glutaraldehyde in 0.1 M PIPES buffer (pH 7.4) for 1 h. The fixative was then replaced with three changes of 0.1 M PIPES buffer. The cells, supported by the coverslips, were then dehydrated in a series of ethanol solutions (at 30, 40, 50, 60, 70, 80, and 90% and then three times at 100%) for at least 20 min for each step. Samples were critical point dried in a Polaron E3000 critical point drier using liquid carbon dioxide as the transition fluid. The coverslips were then mounted with the cell layer facing upward on aluminum scanning electron microscopy (SEM) stubs using sticky tabs. The samples were coated with gold in an Agar high resolution sputter-coater apparatus. SEM was carried out using a Zeiss Supra 55 VP FEG SEM operating at 3 kV.

### Statistics.

Statistical analysis was carried out using both GraphPad Prism and SPSS software. At least three biological replicates (each with three technical replicates) were used to calculate means and the standard errors of the mean. Significance was measured using either Mann-Whitney U test or Bonferroni post test values following analysis of variance (ANOVA).

## RESULTS

### C. jejuni forms increased levels of biofilm in the presence of chicken juice.

**FIG 1:**
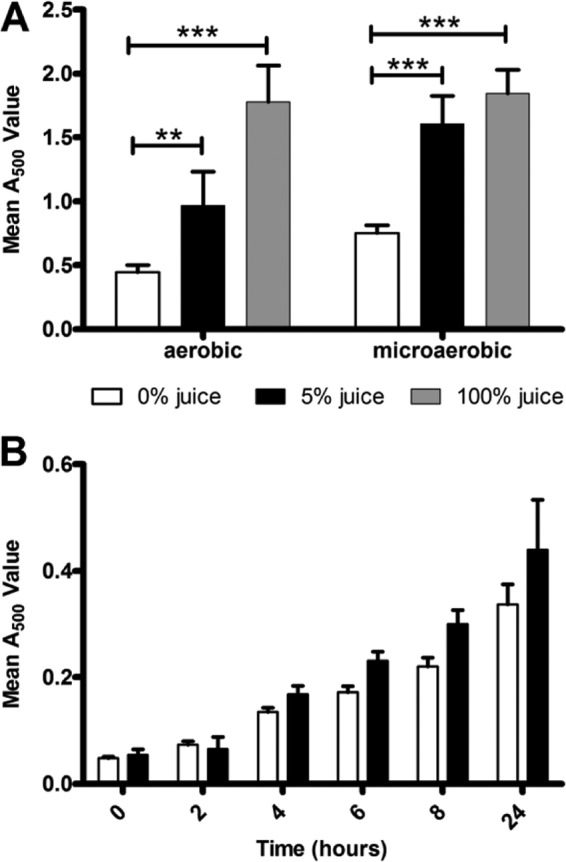
Biofilm formation and growth of C. jejuni NCTC 11168 in the presence of chicken juice. (A) Static incubation of C. jejuni in brucella broth supplemented with chicken juice results in increased biofilm formation, as shown by using a TTC biofilm assay. (B) Growth of C. jejuni in media supplemented with 5% chicken juice is not significantly different from unsupplemented brucella broth. White bars represent unsupplemented brucella broth, and black bars represent brucella broth supplemented with 5% (vol/vol) chicken juice. Error bars show the standard errors of the mean, and significance was measured by using the Bonferroni post test following ANOVA (**, *P* < 0.01; ***, *P* < 0.001).

Meat and meat exudates have been previously reported to allow for an increase in survival of C. jejuni (23, 26, 27). To assess whether meat exudates affect C. jejuni biofilm formation, we measured the biofilm levels in static C. jejuni NCTC 11168 cultures supplemented with meat exudates recovered from defrosted chicken carcasses (chicken juice) and from pork steaks. Since dyes such as crystal violet and Congo red aspecifically bind to meat exudate components (33), we measured biofilm formation by C. jejuni via conversion of the respiratory dye TTC, which relies on detecting redox activity from adhered bacterial cells. Supplementation of brucella broth with chicken juice resulted in an increase in biofilm formation compared to brucella broth alone in both microaerobic and aerobic conditions (Fig. 1A). Replacement of medium by 100% chicken juice gave the highest level of biofilm formation, and this was not due to differences in viability, since cultures incubated in brucella broth, brucella broth with 5% chicken juice, and 100% chicken juice had similar levels of viable planktonic cells. Likewise, addition of pork exudate resulted in a 2-fold increase in biofilm formation in both microaerobic and aerobic conditions (data not shown).

To differentiate between growth and biofilm formation, we assessed growth of C. jejuni NCTC 11168 in brucella broth, brucella broth supplemented with 5% chicken juice, and 100% chicken juice in shaking cultures. There was no statistical difference between growth in brucella broth and media supplemented with 5% chicken juice over a 24 h period (Fig. 1B), and thus the increase in biofilm formation in the presence of chicken juice is likely to be solely due to increased attachment of Campylobacter to the abiotic surface. In 100% chicken juice, the mean *A*_500_ value of the 24 h sample was significantly higher than that of the unsupplemented brucella control (data not shown), suggesting that the increased biofilm formation present in 100% chicken juice could in part be due to enhanced growth of C. jejuni. These results also show that chicken juice supports C. jejuni growth.

### Chicken juice increases biofilm formation in different Campylobacter isolates and on different abiotic surfaces.

**FIG 2:**
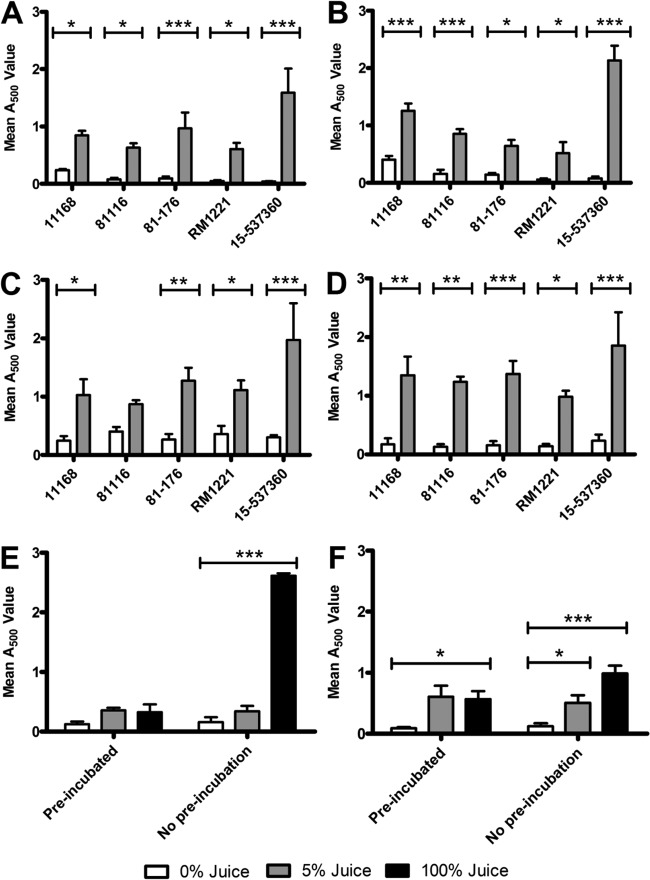
Static incubation of four strains of C. jejuni and one strain of C. coli in the presence of chicken juice leads to increased biofilm formation in various abiotic materials. Graphs A, C, and E show data for biofilms incubated in atmospheric conditions, and graphs B, D, and F show data from a comparable treatment in microaerobic conditions. The materials tested included borosilicate glass (A and B), polystyrene (C and D), and stainless steel (E and F). There was an increase in TTC conversion when brucella broth was supplemented or replaced by chicken juice. The figure shows the quantity of biofilm formation measured by TTC conversion. Error bars show the standard errors of the mean, and significance was measured by using the Bonferroni post test (*, *P* < 0.05; **, *P* < 0.01; ***, *P* < 0.001).

In order to ensure that the effect observed in the glass test tubes was present on other abiotic surfaces and not specific for strain NCTC 11168, we repeated the previous assay using polystyrene plates and stainless steel coupons and extended the assay to three other C. jejuni reference isolates (81116, 81-176, and RM1221) and one C. coli clinical isolate (15-537360). Stainless steel is a commonly used material within the food chain and so is an important surface for bacterial attachment and subsequent biofilm formation and survival. All C. jejuni and C. coli strains showed a significant increase in biofilm formation when brucella broth was supplemented with 5% chicken juice in borosilicate test tubes and 24-well polystyrene wells under both microaerobic and aerobic conditions (Fig. 2A to D). The chicken juice-dependent increase in biofilm formation was particularly clear in C. jejuni RM1221 and C. coli 15-537360, since these strains showed very low levels of biofilm formation in brucella broth alone (Fig. 2A to D). Biofilm formation was also significantly increased in the presence of chicken juice on food grade stainless steel coupons (Fig. 2E and F). Hence, chicken juice is able to promote biofilm formation, independently of Campylobacter isolate or abiotic surface.

### C. jejuni preferentially attaches to chicken juice particulates.

**FIG 3:**
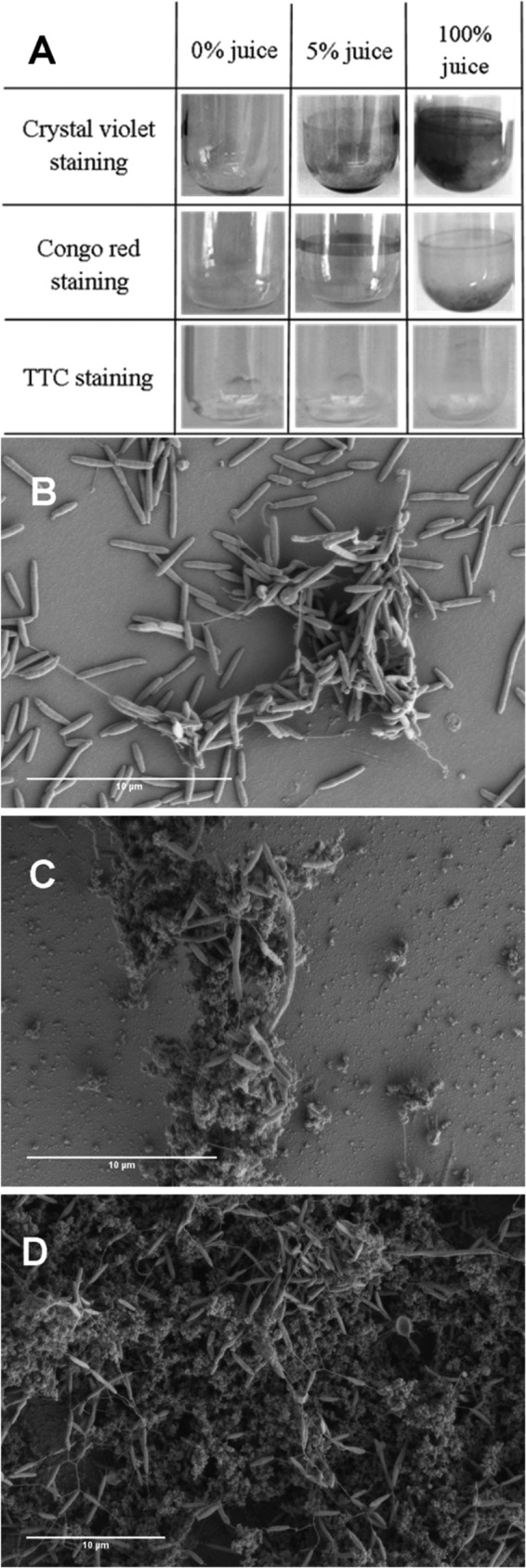
Chicken juice facilitates binding of C. jejuni via modification of abiotic surfaces. (A) Chicken juice components bind to abiotic surfaces such as glass tubes, as shown by crystal violet staining (top row) and Congo red staining (middle row). TTC staining (bottom row) shows that the material bound is not redox reactive. The left column shows results for brucella broth only, the middle column shows results for brucella broth supplemented with 5% chicken juice, and the right column shows results for 100% chicken juice. (B to D) Representative SEM images of C. jejuni biofilms grown in brucella broth supplemented with 0% (B), 5% (C), or 100% (D) chicken juice on coverslips. In chicken juice-containing samples (C and D), C. jejuni can be seen to adhere to the juice particulates rather than the abiotic surface. A large chicken juice particulate can be seen adhered to the slide surface in panel C, with C. jejuni attached to it in preference to the slide surface. In panel D, particulates are densely packed and so cover the field of view. Scale bar, 10 μm.

Since biofilm formation was increased by chicken juice on different surfaces, we investigated the effect of chicken juice on an abiotic surface in the absence of C. jejuni. Brucella broth with or without 5% chicken juice and also 100% chicken juice were incubated in static glass tubes under the standard assay conditions and stained with TTC, crystal violet, or Congo red (Fig. 3A). There was a significant increase in crystal violet and Congo red staining in the presence of chicken juice, while staining with TTC (measuring bacterial respiration) was negative, demonstrating that components of chicken juice bind to the abiotic surface but do not interfere with TTC staining. Since the formation of precipitates (particulates) was also observed, we hypothesized that chicken juice components may form a conditioning layer on the abiotic surface, facilitating bacterial attachment.

In order to further investigate this phenomenon, C. jejuni NCTC 11168 biofilms obtained with brucella broth, brucella broth supplemented with 5% chicken juice, or 100% chicken juice were analyzed by SEM (Fig. 3B to D). In the presence of chicken juice, C. jejuni cells preferentially bind to the particulates rather than directly to the abiotic surface (Fig. 3C and D). This is especially apparent in the 5% chicken juice image (Fig. 3C), where only the chicken juice particulates, but not the abiotic surface, are bound by C. jejuni cells. Figure 3D also visually supports the observations in Fig. 1B that the total number of cells within the biofilm is increased in 100% chicken juice. Hence, chicken juice provides a highly adhesive environment supporting subsequent formation of a C. jejuni biofilm.

### Precoating assay tubes with chicken juice increases biofilm formation.

**FIG 4:**
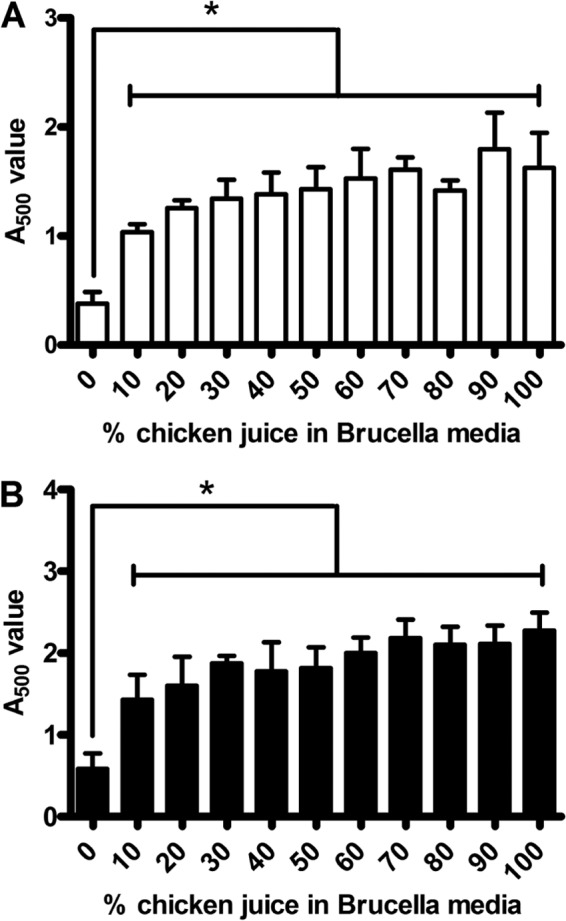
Precoating of test tubes with chicken juice increases biofilm formation by C. jejuni NCTC 11168. Tubes were precoated with a range of chicken juice concentrations before being used in the standard TTC biofilm assay under both aerobic (A) and microaerobic (B) conditions, using unsupplemented brucella broth. Error bars show the standard errors of the mean, and significance was assessed by using the Mann-Whitney U test (*, *P* < 0.05).

All previous experiments were performed with simultaneous addition of C. jejuni and chicken juice, and therefore we investigated whether precoating surfaces with chicken juice also enhanced biofilm formation. A range of chicken juice concentrations was tested during the precoating stage from brucella broth supplemented with 10 to 90% chicken juice and with 100% chicken juice for 24 h at 37°C. Subsequent replacement of precoating medium with C. jejuni NCTC 11168 in unsupplemented brucella broth resulted in a significant increase in levels of biofilm formation with all concentrations of chicken juice compared to brucella broth under both aerobic and microaerobic conditions (Fig. 4). There was no significant increase in levels of biofilm formation with increasing concentrations of chicken juice. This was also observed by precoating stainless steel coupons with chicken juice.

### Chicken juice complements reduced biofilm formation by aflagellated C. jejuni.

**FIG 5:**
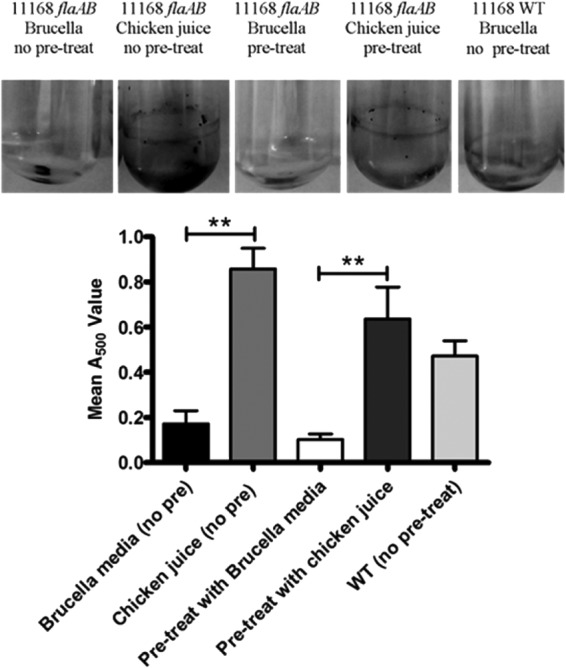
Chicken juice increases the ability of C. jejuni NCTC 11168 Δ*flaAB* mutants to form biofilms in static culture. Static suspensions of C. jejuni Δ*flaAB* mutants were incubated for 48 h to allow biofilm formation in various types of media before TTC staining. A bar chart shows (from left to right) results for C. jejuni Δ*flaAB* mutants in brucella broth (with no pretreatment of the test tubes), C. jejuni Δ*flaAB* mutants in 100% chicken juice (with no pretreatment of the test tubes), C. jejuni Δ*flaAB* mutants in brucella broth (with a 24-h brucella broth pretreatment of the test tubes), C. jejuni Δ*flaAB* mutants in brucella broth (with a 24-h 100% chicken juice pretreatment of the test tubes), and a C. jejuni NCTC 11168 wild-type (WT) culture (with no pretreatment of the test tubes). Error bars show standard errors of the mean, and images above the bar chart are representative of the TTC staining observed for each condition. Significance was measured using a Bonferroni post test following ANOVA (**, *P* < 0.01).

Flagella are known to contribute to attachment and biofilm formation in several bacterial pathogens (34, 35), and an aflagellated C. jejuni Δ*flaAB* mutant produces significantly less biofilm than the wild-type NCTC 11168 strain (10, 36). Incubation with chicken juice or precoating of tubes with chicken juice both resulted in a significant increase of biofilm formation with the C. jejuni Δ*flaAB* mutant compared to incubation in brucella broth alone (Fig. 5). In the presence of chicken juice, biofilm levels were similar to that of wild-type C. jejuni NCTC 11168 (Fig. 5), showing that chicken juice can complement the lack of flagella and support biofilm formation by aflagellated strains. This supports our hypothesis that the effect of chicken juice is mediated through facilitating attachment and not via chemotactic motility.

## DISCUSSION

In this study we investigated the effect of meat exudates on C. jejuni biofilm formation and show that chicken juice is able to enhance biofilm formation compared to brucella broth. Our data show that this is mediated by the ability of chicken juice to provide a conditioning layer on abiotic surfaces, providing an adhesive foundation onto which a C. jejuni biofilm can establish itself and grow. This is observed in both isolates capable of forming biofilms in brucella broth and isolates that are otherwise poor biofilm formers (Fig. 2). In an industrial food setting, this means that the presence of meat exudates can aggravate the problem of contamination by food-borne pathogens such as C. jejuni. Conditioning is defined as the development of absorbed layers onto a surface (22) and can also be seen as biofouling if it is in a undesirable area, for example, the food chain or pipelines (37). Within the food chain, biofouling is an important area of study since it contributes to increased biofilm formation, the loss of heat transfer efficiency, and reduced liquid flow in pipes (37). Our findings add another dimension to the conclusions in a recent literature review on C. jejuni biofilms (38), which concluded that attachment and survival on surfaces and in existing biofilms of other species is the most likely mechanism for C. jejuni to persist in the food chain, rather than *de novo* biofilm formation. Although the aforementioned mechanisms indeed contribute to persistence, meat exudates can enhance survival of C. jejuni by increasing surface adhesion and by providing a scaffold with nutrients and materials the bacterium can use to form a biofilm.

Biofilms are frequently found in the food chain and support bacterial persistence in suboptimal conditions. They are also frequently detected in many different areas of poultry processing plants, from conveyor belts (39) and stainless steel surfaces (25) to floor sealant (40). The food chain is very complex and dynamic, containing varied bacterial contamination sources, environmental conditions, and nutrient sources (41). *In vitro* laboratory studies allow for a reductionist approach, controlling variables to assess the effect of specific conditions, material, or genes on biofilm formation; however, a middle ground must be found in which experimental setup allows control but reflects the complexity of the food chain. The chicken juice system (26) is one method of experimenting with food chain relevance in a laboratory setting. Chicken juice more accurately reflects the conditions in the food chain but is easy to manipulate and reproducible. Several food relevant compounds have been identified to be able to form conditioning layers by their ability to increase biofilm formation in various food relevant bacteria. Bacterial soil increases L. monocytogenes survival on surfaces (21), while milk residues and chicken fillet suspension increase the survival of planktonic Salmonella enterica serovar Enteritidis and C. jejuni on stainless steel (42). Although the notion of conditioning layers within the food chain is not novel, to our knowledge this is the first study proposing a mechanism for the effect of chicken juice on C. jejuni and C. coli biofilm formation, as well as investigating the capacity of chicken juice to condition food chain-relevant abiotic surfaces.

Many animal macromolecules have been reported to be able to form a conditioning film, but they are not always able to enhance biofilm formation. For instance, bovine serum albumin reduces biofilm formation in S. aureus (43) and Burkholderia cepacia (18). Conversely, whey protein and casein are important for Cronobacter biofilm formation (44), although skimmed milk and milk albumin had the opposite effect, inhibiting biofilm formation (22). Additional factors, such as surface roughness and hydrophobicity, will also affect bacterial attachment and biofilm formation. Hydrophilic surfaces, such as stainless steel and glass, increase the time required for bacterial attachment and biofilm formation (37). Surface microstructure is also capable of affecting protein absorption (45), again leading to variability in surface conditioning and subsequent biofilm formation. We have demonstrated that biofilm formation in C. jejuni NCTC 11168 Δ*flaAB* mutants is also enhanced following the precoating of test tubes with chicken juice (Fig. 5), thus complementing the reduced biofilm phenotype of the mutant. This mutant is aflagellated, unable to swarm, and thus unable to migrate toward food sources (46). This means that an increase in attachment and subsequent biofilm formation must be due to alteration of the glass surface properties by the conditioning layer from the chicken juice rather than due to increased chemotactic or energy taxis-directed motility toward a food source.

Since poultry is the most important source of Campylobacter infection in the Western world, we have limited this investigation to the effect that chicken juice has on biofilm formation. Both C. jejuni and C. coli are able to contaminate not only chicken but also turkey, pork, and beef (47). For instance, 49.3% of chicken samples tested were positive for Campylobacter species, along with turkey (37.5% of samples), duck (45.8% of samples), beef (3.2% of samples), pork (5.1% of samples), lamb (11.8% of samples), oysters (2.3% of samples), and milk (1.6% of samples) (48). Subsequent speciation suggested that C. jejuni and C. coli accounted for 83.4 and 16.6% of the isolates, respectively. In our SEM images (Fig. 3B to D), C. jejuni can be observed preferentially attaching to the adhered chicken juice components rather than the surface of the slide. This highlights the need for future studies to not only investigate the link between chicken or pork soil and surface conditioning but also assess the effect of other meat exudates on biofilm formation.

In conclusion, chicken juice allows increased attachment of C. jejuni as it attaches to the surface of the test tubes, providing a conditioned surface for the bacteria to adhere to. This conditioning surface is still present following a simple washing procedure and able to increase biofilm formation if the subsequent incubation with bacteria lacks chicken juice in the broth. Chicken juice also provides a suitable laboratory model for the study of C. jejuni biofilm formation in the food chain, allowing investigators to more closely mimic the food chain conditions that lead to C. jejuni spread and cross contamination of carcasses. Furthermore, identification of the chicken juice components involved in surface conditioning and bacterial attachment may give the opportunity for targeted intervention and prevention strategies to reduce transmission of C. jejuni and C. coli through the food chain.
